# Odontological Society of Pennsylvania

**Published:** 1895-05

**Authors:** 


### ODONTOLOGICAL SOCIETY OF PENNSYLVANIA.

A REGULAR meeting of the Odontological Society of Pennsylvania was held at 1228 Walnut Street, December 8, 1894, Dr. C. N. Peirce in the chair.

After the transaction of routine business, Dr. W. M. Sharp read a paper on "Dental Furnaces."

(For Dr. Sharp's paper, see page 279.)

At the conclusion of the reading the furnace was exhibited.

This furnace is built of clay, and is of sufficient thickness to give volume of heat as well as the necessary temperature. In this respect it is fully equal to the coke or coal furnaces, while all discomfort and dust are avoided.

Besides its great value in continuous-gum work, it can be used for melting zinc, lead, or aluminum, and also affords a ready means of heating a case preparatory to soldering.

Dr. F. A. Coney read a paper on "Carving of Block-Teeth."

(For Dr. Coney's paper, see page 284.)

After both papers had been read, Dr. Peirce, the president, called upon Dr. D. D. Smith, of Philadelphia, to open the discussion.

Dr. Smith responded as follows:

Mr. President and gentlemen, there is little, it seems to me, that can be said upon a subject like this. Thirty years ago, and even later than that, we should have taken a great deal more interest in carving block-teeth. It has been said that there have been no advancements in the line of mechanical dentistry. This seems to me to be a mistake, for there have been a great many improvements made in this branch, and especially in the line of artificial teeth.

For one to be fully skilled as a carver of teeth requires an apprenticeship and experience which very few men are willing to give to it. It does not take a high degree of intellect, but it takes a manipulative finger, which belongs to very few practitioners of the present day.

So far as I know, since Dr. Barstow passed away, there is but one man in the city of Philadelphia that makes any special practice in carving block-teeth. There is scarcely a student but will show you specimens of his carving, but they are simply specimens. They make them possibly in their college course as a matter of curiosity to see what can be done, and immediately on receiving their degrees they forget all about the dirty furnace-work,—for there is a certain amount of dirty work connected with a furnace of any description. I have had at least twenty-five years of solid experience in making continuous-gum work. But my young friend here who read the paper—an admirable paper—cannot tell me a single thing about the management of a furnace. He can tell me a great deal about carving teeth, but it is impossible to make furnace work clean. If you use the old-style furnace, you must go where there is a draught; you must go to the cellar, where the chimney is longest. Now, to go into the cellar a man must don old clothes and overalls, and he must go himself; he cannot send a student or an assistant, if he would have the work done carefully.

The carving of teeth, in practices such as the gentlemen of this city are engaged in, is largely time wasted. Not only on account of the things I have mentioned, but because we can go to our dental depots and get almost everything there. Twenty-five or thirty years ago we could not find the line of teeth in the dental depots that we can find to-day, and when we look them over carefully we see that an incredible amount of thought and care and experience has been bestowed in that direction. Throughout the whole domain of ceramic dentistry there has been a constant upward tendency, and it has not been done by men highly intellectual, but by unskilled labor, with the various steps specialized. For dentists who are educated to-day, who have three years in a dental college, I do not advocate nor believe that it would be well for them to spend their time in the work of carving teeth. The province of the dentist to-day is to combat the diseases of the natural teeth. In a city like Philadelphia there are only about two hundred thousand out of one million who know anything of the benefits of dentistry whatever, except extraction. This is a condition of things that ought not to exist. Though we have three dental colleges filled with dental students and the paraphernalia for advancing the dental profession, still I say that dentistry would take a step backward if all the young men were compelled to give the time to acquire this handicraft and skill.

As the gentleman read the paper I was impressed with the thought of all the manipulations that enamelling teeth involved. He could tell you that it took five years of pretty close practice before he could enamel a set of teeth and get a result that was anyways satisfactory to him the first time.

I know what a difficult thing it was to carve teeth and to crown the *gum-enamel* in such a way as to bring good results after the case was fused; and a dentist can hardly expect to put the enamel on and bring out any such delicate shades as we find in the dental depots at the present time. I do not say but that the teeth are poor enough; there is ample chance for them to be improved, but there has been a great advance in this regard in the manufacture of teeth. We ought to have those who are qualified to do this carving. As dentists it is not necessary for us to do these manipulations, but it is necessary for young men to have a little experience to be able to tell what they want to a mechanical dentist. A single mechanical dentist could make all that we would need in Philadelphia. Why should we devote ourselves individually to this class of work? Let us look to combating the diseases that are incident to the natural teeth.

In regard to the furnace, I would like to see it in operation. The one at the American Dental Association was new; I should say from the looks of this that it had been burned. Have you tested it enough to know whether it will do the work you say it will?

*Dr. Sharp.*—Yes, sir.

*Dr. Smith.*—Every practitioner who cares to do mechanical dentistry can learn now to do continuous-gum work, although it is more difficult than the carving. The results are more uncertain and always will be until we get some different material for the base of continuous-gum work. With this furnace the work is remarkably simplified. This is the first gas furnace that has ever been called to my attention which would do the work. We need volume as well as intensity. We have had intensity but not volume which would be necessary to satisfactorily fuse the material. I have lightened continuous-gum work by using thin plates up to No. 31, so that it would bend in soldering the teeth. About two years ago I sent especially to England for some palladium to get rid of the platinum or heavy metal, for the weight of it lies in the metal largely. I washed the palladium, knowing it was infusible, but I did not know its working qualities under heat. It has the same specific gravity as silver. It has the appearance largely of platinum, a little duller in color when polished, a little more blue. It has more the appearance of aluminum. The palladium necessary for a case cost twenty-two dollars. When sufficient heat was brought upon it to fuse pure gold, at 2012° F., a slight oxidation appeared and the blue color in the polished palladium became tarnished. After soldering the case (it took the solder well) in the repeated fusing the solid material became porous and the oxidation came out very distinctly and colored the body all through with a bluish stain so that I could not overcome it with the enamel. It had not that spotty appearance which the platinum has when it is soldered with alloyed gold. I made the case over with platinum, and that is the best we can do. I think there is no other metal on which you can count for continuous-gum work. You can work No. 29 platinum in connection with continuous-gum work, but thinner than that will cause failure.

*Dr. Truman.*—While I agree with my friend Dr. Smith in his general remarks, and feel that his statement is exactly true that we must aim for something that might be called higher work, still as he has a love for continuous-gum work, so I look back with feelings of pleasure to the time when I had to do mechanical work. I feel that there has been a weakness in dentistry of later years. Ever since metal work was practically abandoned by the general profession we have experienced a loss in the direction of mechanical dentistry, and while bridge-work and crown-work has made it up to some extent, the profession, as such, cannot begin to compare to the days when we were obliged to work gold and silver entirely.

Now, it is a pleasure to me to listen to a paper by a man who comes forward at this late day to advocate block-work. In my recollection as a boy and as a young man, it was one of the most unpleasant parts of dentistry I had to do. As Dr. Smith says, first carve your teeth, biscuit them, etc., then go down into the cellar and burn them, and sit there until that process is accomplished; after that go on day after day and week after week making porcelain teeth. I have no desire to go through it again, yet at the time I had a feeling of pleasure that would almost make me wish to do it again. And I agree with Dr. Smith, if we only had some man who would be willing to do this class of work in conjunction with other kinds of mechanical work exclusively, it would be a great benefit to the profession to-day. As he has stated, there is no one in Philadelphia that does that work. We need them. We cannot always get satisfactory teeth at the depots.

*Dr. Faught.*—I would like to ask Dr. Sharp if the platinum muffle has any advantage?

*Dr. Sharp.*—I have given that some study. Personally I feel that I could get as good results from clay as from platinum. I will give a little demonstration, which I think will be valuable. I have with me a little piece of platinum plate, which I had intended to use in baking the porcelain this evening, but the gas has not been sufficient, and I will use it for this purpose.

In my research or inquiry of the different metals, I find it is a fact that hydrogen will pass through palladium and platinum by diffusion at red heat. This is not so when platinum is cold. I think I will prove this assertion to your entire satisfaction. For that reason I have my doubts about it being superior to clay in continuous-gum work when there is perfect combustion in the furnace. As the hydrogen gas comes in contact with the metal it takes atmospheric oxygen from the atmosphere and produces combustion in the pores of the platinum. If I heat this platinum red hot, and allow it to get partially cooled, and then replace it over the unlighted gas, it will gradually get red and ignite the gas, again showing there is combustion of the gas going on in the pores. (The doctor during this description held the piece of platinum over the gas-jet until it became red, then took it away from the jet and allowed it to resume its natural color; then holding it over the escaping gas again, the flame having been blown out in the mean time, the metal became so hot that the gas was again lighted.) This is good proof that combustion of oxygen is going on in this metal. I don't think there is any advantage whatever in a furnace having a platinum muffle. The disadvantages are that it is very expensive; does not give volume of heat; if the least trace of zinc or lead gets on it by accident, it will be spoiled.

